# Autoimmune Thyroiditis and Glomerulopathies

**DOI:** 10.3389/fendo.2017.00119

**Published:** 2017-06-02

**Authors:** Domenico Santoro, Carmela Vadalà, Rossella Siligato, Michele Buemi, Salvatore Benvenga

**Affiliations:** ^1^Unit of Nephrology and Dialysis, Department of Clinical and Experimental Medicine, University of Messina, Messina, Italy; ^2^Unit of Endocrinology, Department of Clinical and Experimental Medicine, University of Messina, Messina, Italy

**Keywords:** thyroiditis, Hashimoto, glomerulonephritis, membranous glomerulopathy, vasculitis

## Abstract

Autoimmune thyroiditis (AIT) is generally associated with hypothyroidism. It affects ~2% of the female population and 0.2% of the male population. The evidence of thyroid function- and thyroid autoantibody-unrelated microproteinuria in almost half of patients with AIT and sometimes heavy proteinuria as in the nephrotic syndrome point to a link of AIT with renal disease. The most common renal diseases observed in AIT are membranous nephropathy, membranoproliferative glomerulonephritis, minimal change disease, IgA nephropathy, focal segmental glomerulosclerosis, antineutrophil cytoplasmic autoantibody (ANCA) vasculitis, and amyloidosis. Different hypotheses have been put forward regarding the relationship between AIT and glomerulopathies, and several potential mechanisms for this association have been considered. Glomerular deposition of immunocomplexes of thyroglobulin and autoantibodies as well as the impaired immune tolerance for megalin (a thyrotropin-regulated glycoprotein expressed on thyroid cells) are the most probable mechanisms. Cross-reactivity between antigens in the setting of genetic predisposition has been considered as a potential mechanism that links the described association between ANCA vasculitis and AIT.

## Introduction

Hashimoto’s thyroiditis is the leading form of autoimmune thyroiditis (AIT), which is the most prevalent autoimmune disorder and the most common cause of hypothyroidism, excluding iodine insufficiency. It affects ~2% of the female population and 0.2% of the male population (1). This condition is well known to be associated with other autoimmune diseases, the most common of which are chronic autoimmune gastritis, vitiligo, rheumatoid arthritis, polymyalgia rheumatica, celiac disease, type 1 diabetes, Sjögren’s syndrome, systemic lupus erythematosus (SLE), multiple sclerosis, and sarcoidosis (2). Also glomerular disease may be related to autoimmune disease with several mechanisms.

## The Effects of Thyroid Hormones on Kidney

Thyroid hormone influence on kidney is mediated by its effect on the cardiovascular system and, consequently, by its effect on renal blood flow. Hypothyroidism initially decreases peripheral vascular resistance and blood pressure and subsequently activates the renin–angiotensin–aldosterone system, which increases tubular sodium reabsorption. As a consequence, cardiac preload and vascular resistance raise, resulting in increased diastolic blood pressure and cardiac afterload (3). Renin gene expression is also regulated by circulating levels of free triiodothyronine (FT3) and free thyroxine (FT4) through beta-adrenergic activation; accordingly, the reduced sensitivity to beta-adrenergic stimulus occurring in hypothyroidism can cooperate with other hemodynamic abnormalities decreasing renin release (4, 5). The resulting negative inotropic effect on the heart, as well as the altered equilibrium between the reduced expression of vasodilators such as vascular endothelial growth factor or insulin-like growth factor-1, can lead to further renal vasoconstriction. Other consequences of thyroid hormone deficiency include lower secretion of atrial natriuretic factor and erythropoietin, therefore reducing further blood volume (6). Glomerular filtration rate (GFR) can thus decrease by up to 40%, with subsequent elevation of serum creatinine, both indices being directly proportional to circulating TSH levels (and therefore directly proportional to the extent of thyroid failure) independent of other confounding factors such as age, sex, body mass index, or comorbidities. Thyroid hormone replacement in patients with overt or subclinical hypothyroidism restores renal function (4, 7–9).

Experimental models of hypothyroid mice show kidney hypotrophy and altered glomerular structure (6). Salomon and colleagues studied the histopathology of renal lesions in a group of seven patients with hypothyroidism of both primary and secondary etiology (10). They discovered a common pattern in the renal biopsies from all seven patients, which was directly proportional to the duration of disease (10). Electron microscopy highlighted the thickening of both glomerular and tubular basement membranes, due in part to widening of the dense layer (*lamina densa*) and in part to considerable enlargement of the inner light layer (*lamina rara interna*); mesangial matrix was increased. Glomerular cells (epithelial, endothelial, and mesangial) presented a variety of osmiophilic inclusions, most of them containing lipid. Tubular cells contain similar inclusions and also homogenous protein reabsorption droplets (10).

Gao et al. (11) measured serum β2-microglobulin, urine β2-microglobulin, albumin, and immunoglobulins in 39 untreated AITD (28 with Graves’ disease and 11 with Hashimoto’s disease). Microproteinuria was found in 28.6% of patients with Graves’ disease and in 45.5% with Hashimoto’s disease. Serum β2-microglobulin concentrations were significantly increased in Graves’ disease compared with that of controls. They concluded that the renal lesions associated with AIT are present in both the glomerulus (leading to increased glomerular capillary permeability) and the tubulus.

Free triiodothyronine can also influence the expression of structural and regulatory proteins in renal tubuli, particularly Na^+^/K^+^ ATPase and Ca^2+^ and Na^+^/H^+^ exchanger, which have a reduced activity in animal models of long-term hypothyroidism (12). These animals also have increased urinary excretion of sodium and bicarbonate, and defective urinary acidification. Lower medullary hypertonicity results in impaired urinary concentrating ability. On the other hand, increased sensitivity to vasopressin can stimulate water reabsorption (13). Moreover, the filtrate overload caused by altered tubular reabsorption processes, as well as the dysregulation of chloride channels ClC-2, are responsible for the activation of tubuloglomerular feedback, which has important effects on GFR (14). Another important feature of hypothyroid murine models was the increased vascular calcification related to the lower expression of the matrix Gla protein, which physiologically exerts a protective role on vascular calcification (15).

## Mechanisms of Autoimmunity in Kidney Disease

Kidney can be the victim of autoimmune processes through several mechanisms. Autoantibodies can damage glomeruli either targeting specific antigens as in membranous glomerulonephritis (16) and in anti-glomerular basement membrane (GBM) nephropathy (17), or being trapped through the filtration barrier as in antineutrophil cytoplasmic autoantibody (ANCA) vasculitis (18) or IgA nephropathy (19). Pathophysiology of renal impairment in the course of SLE is characterized by both events, because anti-DNA antibodies are located in capillary membranes and mesangial areas of glomeruli and because they cross-react with α-actinin and glycosaminoglycans on mesangial cells (20). All these immune complexes alter the structure of basement membrane, podocyte function, and activate the classical pathway of complement system, which exacerbate the inflammatory process due to chemotactic factors C3a and C5a. In addition, terminal pathway of complement worsens cell damage because of the cytolytic effect of C5b-9 complex (21). Finally, immune complexes stimulate infiltration of innate and specific immune cells, such as neutrophils, macrophages, natural killer (NK) cells, and T lymphocytes, which express receptors for constant fraction (FcR) (22, 23).

Natural killer cells have also a role in the pathogenesis of kidney damage as they produce interferon γ (IFNγ) and activate peripheral macrophages first and, then, resident glomerular cells that are responsible of chronic processes (24, 25).

Kidney-resident dendritic cells secrete IL-23 to recruit both γδ T cells, a specific T subset with adaptive and innate features and a pro-inflammatory role consisting in regulatory T cells (T_reg_) inhibition, stimulation of B lymphocyte antibodies production and the secretion of cytokines (26). In particular, γδ T cells and double-negative CD4^−^CD8^−^ T cells sustain production of IL-17, which is responsible for neutrophils recruitment. They, in turn, have a central role in damaging kidney through the programmed cell death of neutrophil extracellular traps (NETosis) and the production of reactive oxygen species that stimulate mesangial cell proliferation and cytotoxicity mechanisms (27–29). In addition, IL-17 promotes expression of C-C motif chemokine 20 (CCL20) on mesangial cells, that recruit T helper cells producing IL-17 (T_H_17). T_H_17 are able to maintain kidney damage and promote B-cell activation through the secretion of IL-21 (30). Finally, T follicular helper cells, a subpopulation of CD4^+^ that is increased in autoimmune processes, act as stimulator of B cell differentiation into plasma cells (31–33).

T_H_1 lymphocytes and IFNγ stimulate macrophage recruitment in SLE- and ANCA-associated vasculitis, as well as anti-GBM nephropathy in experimental models. However, the role of T_H_1 lymphocytes in human autoimmune renal disease is not clearly defined yet (34–36). CD8^+^ T cells have a pathogenic role in ANCA-associated vasculitis, since they can produce both IFNγ and tumor necrosis factor (37). The presence of CD8^+^ T cells is also correlated with poor prognosis (38).

Regulatory T cells as well as NKT cells act as regulators of immune response and are reduced in autoimmune disease (39, 40).

## Glomerular Disease Related to AIT

**Figure 1:**
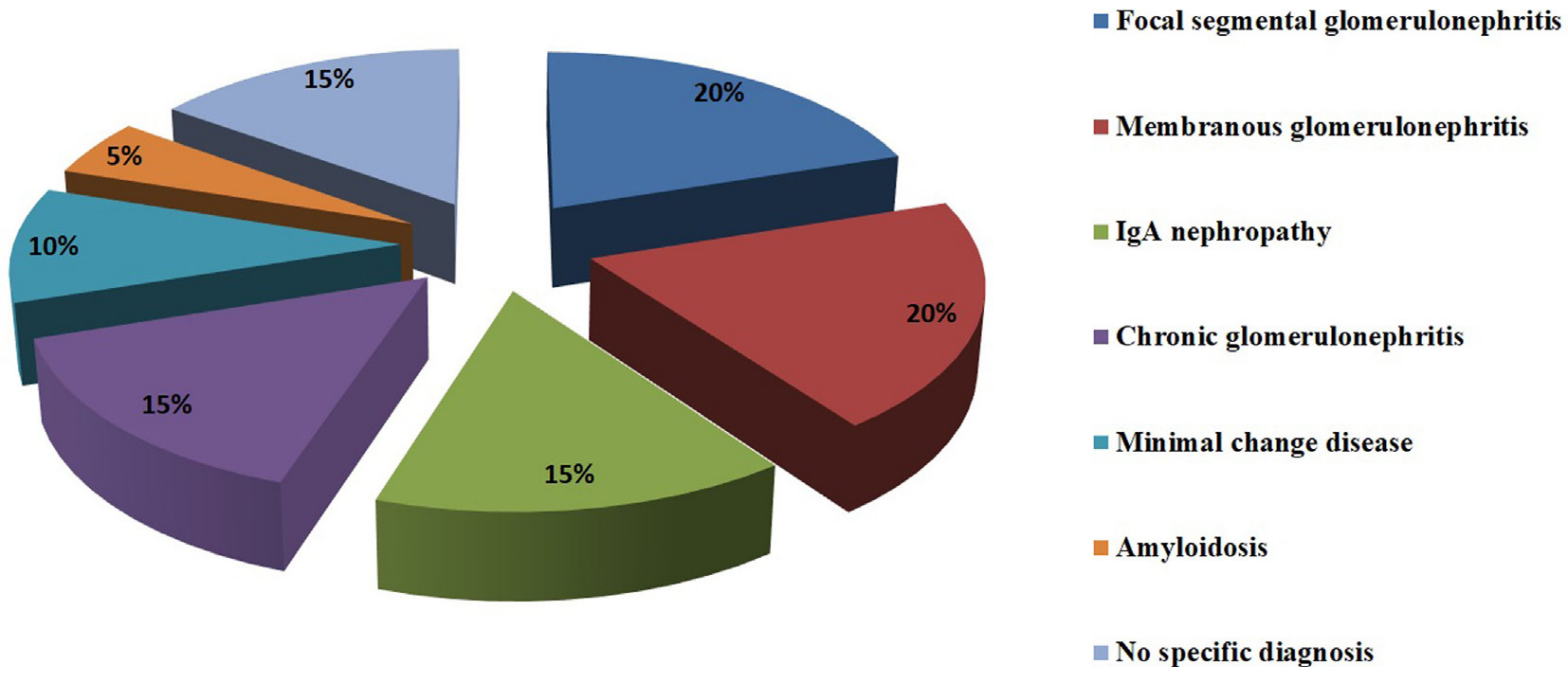
Glomerulopathies associated with HT.

Glomerular involvement in patients with AIT can occur in 10–30% of cases (41). A retrospective study on 28 patients with Hashimoto’s thyroiditis and hematuria, proteinuria, or renal impairment showed that the most common associated kidney diseases are membranous glomerulonephritis (20%), focal segmental glomerulosclerosis (20%), IgA nephropathy (15%), chronic glomerulonephritis (15%), minimal change disease (10%), and amyloidosis (5%). In 15% of the 28 patients, no specific diagnosis was made (42). Other case reports revealed the less frequent connection between AIT and membranoproliferative glomerulonephritis and ANCA vasculitis (43–47) (Figure 1).

Various hypotheses were considered to explain the underlying mechanism that links AIT to glomerular lesions and their variable presentation (Table 1).

**Table 1:** Mechanisms underlying the relationship between HT and kidney disease.

*In situ* immune response against thyroglobulin (TG) deposition at subepithelial level
Circulating TG–anti-TG complexes trapped at subendothelial level due to increased glomerular permeability
Megalin (gp330) as a possible immunologic target
Epitope spreading
Genetic predisposition and cross-reactivity between antigens

The higher prevalence of membranous nephropathy (MN) suggests a plausible immunologic role of thyroid antigens, particularly thyroglobulin (TG) and thyroperoxidase (TPO). Both of them are released in the course of AIT and are found in subepithelial immune deposits, as part of the characteristic spikes of MN (47, 48). At present, there are two possible mechanisms that can explain the immunologic role of thyroid antigens in the pathogenesis of MN: (1) *in situ* immune response against TG deposition at subepithelial level and (2) circulating immune complexes (TG–anti-TG) that can be trapped at subendothelial level due to increased glomerular permeability. As stated before, the pathogenicity of immune complexes in MN is related to their subepithelial localization, but how they could cross GBM remains unexplained. Most likely, immune complexes could dissociate in the subendothelial space and then they would reassemble on the subepithelial side. IgG4 is considered the main antibody subclass deposited in the course of idiopathic MN. Specific subclass of anti-TG and anti-TPO antibodies should be determined in patients with suspicious AIT-related glomerulopathy to distinguish between a clear diagnosis of idiopathic MN or a possible IgG4-mediated secondary form of MN. Moreover, IgG4 antibodies have low affinity for the antigen, which could explain the possible dissociation and reassociation of the IgG4 complexes through the GBM (49).

Other theories involve the mechanism of epitope spreading, a phenomenon that follows the primary immune response against specific epitopes. When the immunodominant response fails to clear the target, the immune system mounts a broader inflammatory response against different epitopes either on the same or on different molecules. Therefore, immune-mediated glomerular disease would be caused by a subset of autoantibodies directed toward epitopes of TG or TPO as well as epitopes of glomerular antigens. This phenomenon may be relevant to the pathogenesis of kidney disease, since in Heymann nephritis (a murine experimental model of membranous glomerulonephritis) the onset of proteinuria correlates with intramolecular epitope spreading (50). In addition, epitope spreading has already been demonstrated in experimental immunization with an immunogenic TG peptide, but has not been investigated in patients yet (51).

The experimental Heymann model also suggests megalin (gp330) as a possible immunologic target involved in the immunopathogenesis of glomerular injury during AIT. Megalin is a large glycoprotein receptor expressed on thyrocytes in a TSH-dependent manner, but it is also expressed on the renal proximal tubular cells (52). Megalin is a receptor that interacts with various intracellular adaptor proteins for intracellular trafficking and that functions cooperatively with other membrane molecules (52). Megalin is involved in the uptake of glomerular-filtered albumin and other molecules such as insulin, hemoglobin, vitamin D-binding protein, retinol-binding protein, and β_2_-microglobulin. In addition, a number of toxic substances, such as glycated proteins (AGEs), myeloma light chain, and aminoglycosides, undergo megalin-mediated endocytosis, leading to cell damage (52). AIT could determine a rupture of immune tolerance toward this self-antigen, thus causing an immune response on podocytes.

The relationship between AIT and ANCA vasculitis was shown by Lionaki and colleagues (53). In their case–control study, they demonstrated that when ANCA vasculitis was diagnosed, as many as 40% of women had thyroid disease. Among men, the prevalence of thyroid disease was lower. Patients with positive anamnesis for thyroid disease were more likely to have myeloperoxidase (MPO)-ANCA (86%) than proteinase 3-ANCA (14%) (53). Both genetic predisposition and cross-reactivity between antigens have been hypothesized as potential mechanisms for this association. A functional polymorphism in the protein tyrosine phosphatase gene, the *PTPN22* 620W allele has been recognized as a predisposing factor for several autoimmune disorders, including AITD, Wegener’s granulomatosis, and ANCA positivity (54–56). *PTPN22* is located on chromosome 1p13.3–13.1.10 and encodes an 807-amino acid protein that interacts with Csk, a tyrosine kinase that is involved in the intracellular signaling cascade following T-cell activation. A missense variation in the autoimmunity-predisposing allele results in gain of function that increases the threshold for T-cell receptor signaling (57). As in other multifactorial processes, one or more environmental triggers are necessary for the full development of the disease. Occupational exposures to factors such as silica (58) showed an association with ANCA vasculitis, while infections such as *Yersinia enterocolitica* or retroviruses have been postulated to participate in the pathogenesis of AITD (56). Eventually, cross-reactivity between TPO and MPO may be another mechanism involved in the development of autoimmunity, due to the strong homology between amino acids 586–601 of TPO and amino acids 594–609 of MPO (59, 60).

Type 1 diabetes mellitus (DM1), a known autoimmune disease that can be present in 3–8% of patients with Hashimoto’s thyroiditis (61) and in 6–10% of subjects with Graves’ disease (62), is worth mentioning at this point. Benvenga et al. investigated the presence of serum antibodies directed against one or both thyroid hormones (THAbs), which are considered to be rare autoantibodies, in a cohort of 52 DM1 patients both at baseline and after 6 years of follow-up (63). They found that serum THAb could be predictive for concurrent or subsequent DM1-related complications, including diabetic nephropathy. Patients already affected by nephropathy showed either T3IgG or T4IgM at baseline. T4IgM was associated with a high rate of retinopathy (67%), nephropathy (50%), and neuropathy (33%). At tissue level (kidney, in this particular case), THAb may decrease the local availability of thyroid hormones, not much differently from the decreased tissue availability resulting from the decreased thyroid output of thyroid hormones. Clearly, this study (63) awaits confirmation by future investigations.

Finally, renal diseases presenting as nephrotic syndrome can lead to the onset or the aggravation of preexisting hypothyroidism. The urinary loss of both protein-unbound (free) and protein-bound thyroid hormones, with consequent decreased serum levels of T4, T3, FT4, FT3, and major carrier proteins (thyroxine-binding protein, transthyretin, and albumin), is directly proportional to proteinuria. The practical consequence of this urinary loss is the increased requirements of the daily L-T4 replacement (64).

## Conclusion

A relationship between AIT and glomerulonephritis does exist, but it requires further investigations in larger cohorts. A common pathogenesis may be considered, especially in patients with simultaneous appearance of glomerular and thyroid dysfunction. Monitoring kidney function should be considered as part of the follow-up of AIT patients, particularly of those with HT-related hypothyroidism.

## Author Contributions

Conceptualization: DS, CV, RS, and SB. Research literature: DS and MB. Methodology: DS and RS. Supervision: DS, MB, and SB. Writing original draft: DS, CV, and RS.

## Conflict of Interest Statement

The authors declare that the research was conducted in the absence of any commercial or financial relationships that could be construed as a potential conflict of interest.
